# Amlodipine, an L-type Ca^2+^ channel inhibitor, regulates release of extracellular vesicles from tumor cells

**DOI:** 10.1093/carcin/bgaf016

**Published:** 2025-03-23

**Authors:** Sujan K Mondal, Chang-Sook Hong, Jie Han, Brenda Diergaarde, Dan P Zandberg, Theresa L Whiteside

**Affiliations:** Department of Pathology, University of Pittsburgh School of Medicine, UPMC Hillman Cancer Center, 5117 Centre Ave, Suite 1.32, Pittsburgh, PA 15213, United States; Department of Pathology, University of Pittsburgh School of Medicine, UPMC Hillman Cancer Center, 5117 Centre Ave, Suite 1.32, Pittsburgh, PA 15213, United States; Department of Pathology, University of Pittsburgh School of Medicine, UPMC Hillman Cancer Center, 5117 Centre Ave, Suite 1.32, Pittsburgh, PA 15213, United States; Department of Human Genetics, School of Public Health, University of Pittsburgh, UPMC Hillman Cancer Center, 5117 Centre Ave, Pittsburgh, PA 15213, United States; Department of Medicine, University of Pittsburgh School of Medicine, UPMC Hillman Cancer Center, 5117 Centre Ave, Pittsburgh, PA 15213, United States; Department of Pathology, University of Pittsburgh School of Medicine, UPMC Hillman Cancer Center, 5117 Centre Ave, Suite 1.32, Pittsburgh, PA 15213, United States; Departments of Immunology and Otolaryngology, University of Pittsburgh School of Medicine, UPMC Hillman Cancer Center, 5117 Centre Ave, Suite 1.32, Pittsburgh, PA 15213, United States

**Keywords:** cancer, extracellular vesicles, amlodipine, tumor growth, autocrine effects

## Abstract

Tumor cells produce/release tumor-derived exosomes (TEX) which promote tumor growth, drive immune suppression, and interfere with immune therapies. Amlodipine, a calcium flux inhibitor, may block TEX release by tumor cells. Amlodipine’s potential as a drug blocking TEX release was evaluated. We measured tumor growth, TEX numbers, phenotype, and molecular content in murine SCCVII and human cancer cell lines. Cell lysates and TEX were tested for expression of autophagy-related proteins by western blots (WBs). Tumor growth in mice, histopathology, T-cell infiltrations, and TEX production by SCCVII treated with amlodipine were measured. Numbers and protein content of TEX eluted from tumor explants were studied by flow cytometry and WBs. Amlodipine used *in vitro* at 0.5–5 µM was nontoxic, did not impair tumor cell viability, reduced cell proliferation, and decreased TEX production. It reduced PD-L1 and Rab11 content of TEX, altered tumor cell size/shape, induced vesicle accumulations in the cytosol, and upregulated expression levels of autophagy-related proteins, ATG7, Beclin-1, and LC3. *In vivo*, daily treatment of established SCCVII with amlodipine (10 mg/kg) inhibited tumor growth (*P* < 0.001), increased CD8^+^ T-cell infiltration into tumor, decreased TEX production, and altered PD-L1, Rab11, and FasL content of TEX. Amlodipine delivered *in vitro* to tumor cells or *in vivo* to tumor-bearing mice interferes with tumor growth and TEX production, induces tumor autophagy, reduces circulating TEX numbers, and alters the TEX immunosuppressive signature. Amlodipine emerges as a potentially promising drug for removing immunosuppressive TEX in cancer subjects who are candidates for immune therapies.

## Graphical Abstract

**Graphical Abstract:**
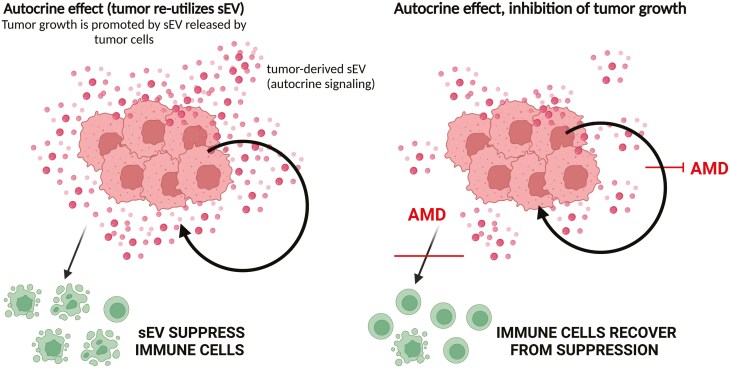

## Introduction

Small extracellular vesicles (sEVs) produced by tumor cells are referred to as tumor-derived exosomes or TEX. These vesicles regulate tumor growth and modulate functions of the host immune system (1–4). Most studies of TEX were performed using sEV isolated from supernatants of tumor cells, where all vesicles are tumor derived. Injected into mice, murine or human TEX circulate freely in plasma and body fluids crossing all tissue barriers (5,6). TEX represent a communication system between tumor and nonmalignant cells (1). Body fluids of cancer patients contain a heterogenous mix of TEX and of EVs derived from nonmalignant cells. These EV subsets have distinct immunosuppressive protein profiles (7,8). Unlike sEV produced by healthy cells, TEX promote tumor growth and induce dysfunction or death of immune effector cells *in vitro* and *in vivo* (9,10). TEX carry immunosuppressive proteins and miRNAs, and their presence and abundance in plasma of cancer patients correlate with clinicopathological endpoints and disease activity (11,12).

Tumors in mice and humans produce and release biologically active TEX in numbers that significantly exceed those in plasma of healthy subjects (13,14). Circulating sEV in plasma of cancer patients are enriched in TEX (15). In melanoma, the numbers of circulating TEX carrying on their surface immunosuppressive receptor/ligands have been linked to advanced disease (16). Therefore, a removal from plasma or silencing of negative (i.e. immunosuppressive) signaling by TEX could represent a potentially effective therapeutic anti-cancer strategy. In hosts bearing tumors, a reduction in numbers/activity of circulating sEV enriched in TEX could deprive the tumor of the autocrine growth supporting mechanism TEX provide and could lead to the recovery of anti-tumor immunity. A reduction in levels of TEX in plasma could be obtained by removal of sEV from the circulation, e.g. by therapeutic plasma exchange (TPE) or by suppression of sEV production by the tumor. However, a removal by dialysis of circulating sEV from plasma does not discriminate TEX from EVs produced by nonmalignant cells, is strictly dependent on the balance of sEV production/sEV removal (17), and may not be as effective as expected. A recent clinical study designed to clear circulating PD-1^+^ and PD-L1^+^ EVs in patients with melanoma demonstrated a limited clinical efficacy of this strategy (18). Alternatively, inhibition of TEX production by the tumor might be a more effective approach to selective blocking of TEX pro-tumor activities. To this end, a report that amlodipine (AMD), a calcium channel blocker (CCB) and a commonly used hypertension drug, downregulated the release of sEV from tumor cells (19)prompted us to advance the hypothesis that AMD blocks TEX release by human cancer cells *in vitro* and murine cancer cells *in vivo*, thereby suppressing tumor growth and promoting recovery of anti-tumor immunity. Here, we show that selective inhibition of immunosuppressive TEX production/release by AMD emerges as a potentially effective immuno-restorative therapeutic strategy for tumor-bearing hosts.

## Materials and methods

### Cell lines and culture

#### Human HNSCC cell lines

PCI-13 was established, authenticated, and maintained at the Whiteside laboratory, University of Pittsburgh (20), and SCC47 cell line was obtained from Dr T. Carey, University of Michigan, banked, and authenticated prior to culture at the University of Pittsburgh Cytogenetics Institute. Murine SCCVII cell line was obtained from Dr R.J. Wong (21), banked, and a fresh aliquot of cells was drawn from the cell bank and cultured when needed for this study. All cultures were mycoplasma free. Cryopreserved cells were thawed, washed, plated in culture flasks, and grown at 37°C in the atmosphere of 5% CO_2_ in air. Cells were cultured in RPMI1640 medium, 1% (v/v) penicillin/streptomycin, and 10% (v/v) heat-inactivated fetal bovine serum (FBS; ThermoFisher Scientific) depleted of EVs by ultracentrifugation at 100 000 × *g* for 3 h. Cells were passaged when they reached 70% confluence.

For vesicle isolation from supernatants, cells were cultured in 150 cm^2^ cell culture flasks containing 25 ml of the culture medium. Each flask was seeded with 4 × 10^6^ cells, and following 72 h of incubation, supernatants were collected, while the cells were harvested using 2 ml TrypLE Express (Gibco) and washed in serum-containing medium. For subsequent passages, cells were re-seeded in new flasks using the cell numbers described above. Supernatants were collected for sEV isolation.

### Isolation of sEVs from supernatants of cell lines

Cell culture supernatants were combined, and a 50 ml aliquot of cell culture supernatant was centrifuged at room temperature (RT) for 10 min at 2000 × *g* to sediment cells and cell fragments. Supernatants were transferred to new tubes for centrifugation at 10 000 × *g* at 4°C for 30 min. Supernatants were collected and filtrated using a 50 ml syringe and a 0.22 µm bacterial filter. Afterwards, aliquots of supernatants were concentrated to 1 ml using Vivacell 100 concentrators (Sartorius Corp.) at 2000 × *g* (22).

### sEV isolation and characterization

sEVs were isolated by size exclusion chromatography (SEC) as described by us (23). An aliquot (1 ml) of concentrated supernatant was loaded on a 10-cm-long Sepharose 2B column and was eluted with phosphate-buffered saline (PBS). Individual 1 ml fractions were collected. Fraction #4 containing the bulk of non-aggregated morphologically intact sEVs was harvested, concentrated using 100 000 MWCO Vivaspin 500 centrifugal concentrators (Sartorius Corp.), and evaluated for protein, vesicle size, molecular content, and sEV functions (23). Transmission electron microscopy (TEM) was performed at the Center for Biologic Imaging, the University of Pittsburgh. The concentration and size distribution of sEVs were measured by nanoparticle tracking analysis (NTA) using NanoSight 300 (Malvern, UK).

### Western blot analysis

To concentrate isolated sEVs, 0.5 ml 100K Amicon Ultra centrifugal filters (EMD Millipore) were used for centrifugation at 4000 × *g*. Vesicle aliquots were lysed with Laemmli sample buffer (Bio-Rad Laboratories, Hercules, CA, USA) and separated using 4-15% sodium dodecyl sulfate–polyacrylamide gel electrophoresis (SDS–PAGE) gels. Each lane was loaded with 10 μg of fraction #4 protein. After transfer from gels to the polyvinylidene fluoride (PVDF) membranes, proteins were detected using antibodies (Abs) specific for selected proteins. Abs used for western blots (WBs) are listed in Supplementary Table 1.

### Functional activity of sEVs

The ability of isolated sEVs (10 µg protein) to induce apoptosis of CD8^+^ Jurkat T cells during a 6-h coincubation was measured by flow cytometry using FITC Annexin-V (ANXV) Apoptosis Detection Kit (BD Biosciences, #55647) in a Cytoflex flow cytometer (Beckman) as previously described (9).

### *In vitro* effects of AMD on tumor growth

AMD was purchased from Cayman Chemical (cat. #14838) and dissolved in 1 ml of dimethylsulfoxide (DMSO). PBS was added to obtain the AMD stock solution at 10 μM. Cells or sEVs were co-incubated with AMD adjusted to the final concentrations ranging from 0.1 to 5 µM as specified in each coincubation experiment. To measure SCCVII cell proliferation, 2 × 10^4^ SCCVII cells were plated in wells of 24-well plates and cultured in RPMI media without serum overnight. sEVs (2.5 or 5.0 µg) were added to triplicate wells and cells were cultured for 48 h. Cell proliferation was measured using a CCK-8 kit (96992, Sigma-Aldrich, St Louis, MO, USA). Positive controls were cultured in medium containing 10% (v/v) FBS. Negative controls contained PBS in place of sEVs. Cell viability was measured by performing cell counts in the presence of the trypan blue dye. To prepare cell lysates for WBs, 1 × 10^6^ SCCVII cells were plated in wells of 6-well plates and co-incubated with different concentrations AMD (2.5 or 5.0 µM) or PBS/DMSO for 24 h prior to lysis in RIPA lysis buffer (89900, ThermoFisher, Waltham, MA, USA).

### Production of GFP^+^ sEVs by SCCVII cells

Tumor cells expressing CD63-GFP fusion protein were generated by stable transfection of plasmid pHluorin-M153R-CD63-mScarlet (plasmid #172118, Addgene, Watertown, MA, USA) as recommended by the plasmid manufacturer. Lipofectamine (2 µl; L3000001, Invitrogen, Carlsbad, CA, USA) was mixed with 1 µg of plasmid DNA, and the DNA and lipofectamine complex was incubated with 40 000 cells in OptiMEM medium for 4 h. The cells were cultured for 48 h in complete medium and then were cultured for 4 days with 1.5 µg/ml blasticidin (Sigma) in RPMI containing 10% FBS depleted of exosomes. Tumor cells producing strong mScarlet (red) staining under a fluorescent microscope were selected and expanded. Supernatants were collected and tested for the presence of GFP^+^ sEVs using a fluorescent microscope.

### EV uptake imaging

sEVs (10 µg protein) isolated from culture supernatants of SCCVII cells transfected with the pHluorin-M153R-CD63-mScarlet were co-incubated with autologous SCCVII (1 × 10^5^ cells) for 12 h. Tumor cells expressing GFP in the cytosol were imaged using Amnis Image Stream Flow Cytometer.

### Confocal microscopy

Indirect immunofluorescence of SCCVII cells was performed using rabbit anti-Rab7 Ab and donkey anti-rabbit IgG-FITC. Actin was stained with F-actin-rhodamine-phalloidin (Supplementary Table 1). Confocal microscopy was performed at the Center for Biologic Imaging, University of Pittsburgh.

### *In vivo* effects of AMD in mice

#### Mice

Several different mice groups were purchased from The Jackson Laboratory (Bar Harbor, ME, USA) at different time points during this 2-year study as follows: Cohort 1, C3H-HeJ mice (female; healthy, no tumor implantation); Cohort 2, C3H-HeJ mice (female; intravenous [IV] tumor implantation); Cohorts 3 and 4 C3H-HeJ mice (female; subcutaneous tumor established prior to AMD injections); and Cohort 5 C3H-HeJ mice (male; subcutaneous tumor established prior to AMD injections).

Protocols for animal experiments were approved by the Institutional Animal Care and Use Committee (IACUC) under the protocol #18042580. On arrival to the Animal Facility, the mice were 4–6 weeks old. They were randomly divided into groups for further treatment (each group = 5–12 mice). Mice were housed in cages on autoclaved laboratory animal bedding. All mice had *ad libitum* access to water. After an initial 1-week adaptation period, mice were used for the respective studies.

#### AMD treatment dose preparation for injections into mice

Due to low AMD solubility in aqueous buffer, 8.5 µl AMD (228 mg/ml) in DMSO was first mixed with 8.5 µl of Cremophor oil, and then this mixture was diluted with 683 µl PBS to prepare 0.7 ml of the AMD treatment solution. For vehicle control, a similar dose was prepared without AMD. Both the vehicle control and the AMD dose were injected into mice intraperitoneally (IP) as described below.

#### AMD treatment of healthy mice (Cohort 1)

To evaluate the potential toxicity of AMD, healthy 4- to 6-week-old C3H-HeJ mice were randomly divided and injected daily with vehicle (*n* = 5) or AMD 10 mg/kg (*n* = 5) for 2 weeks. Blood was collected on days 0 (before start of AMD injection), 4, 7, 14, and 18 from mouse submandibular vein using 4 mm lancet, and blood was placed in a heparinized tube (Sarstedt #16.443.100) for subsequent processing to isolate and count sEVs.

#### Tumor models

In the *lung metastasis model (Cohort 2)*, 4- to 6-week-old female C3H-HeJ mice were injected with 1 × 10^5^ SSCVII cells in 100 µl PBS via the tail vein. After 4 days, mice were randomly divided into two groups: vehicle control (*n* = 5) and treatment cohort (AMD at 10 mg/kg, *n* = 5). Vehicle and AMD were injected IP every day starting on day 4 until day 24. On day 25, the mice were sacrificed, and blood was collected by cardiac puncture using a syringe for sEV isolation. The lungs were harvested, photographed, and processed for histopathological analysis.

In the *subcutaneous tumor model (Cohorts 3 and 4)*, 2 × 10^6^ SCCVII cells in 100 µl PBS were subcutaneously (sc) injected in the right flank of 4- to 6-week-old female C3H-HeJ mice. For *Cohort 3*, mice were divided into two groups: vehicle (*n* = 5) and AMD at 10 mg/kg (*n* = 5) on day 13 following tumor cell injection. Mice in *Cohort 4*, were treated either with vehicle (*n* = 12) or AMD at 10 mg/kg (*n* = 12) or AMD at 15 mg/kg (*n* = 12) starting on day 5 following tumor cell injection. IP treatments were performed daily until day 27 for Cohort 3 and until till day 21 for Cohort 4. The tumor growth was monitored in each mouse every 3–4 days by measuring the longest and shortest dimension of the tumor using a vernier caliper. The tumor volume was calculated as 0.5 × *a* × *b*^2^, where *a* and *b* are the longest and shortest tumor dimensions, respectively. At various time points, blood was collected from the submandibular vein using 4 mm lancet and placed in a heparinized tube for subsequent sEV isolation and counts. Cohort 3 mice were sacrificed on day 28 and Cohort 4 mice on day 22. Tumors were excised, photographed, and weighted, and tumor tissues as well as spleens were processed for histological and immunological analyses. *Cohort 5* consisting of 10 C3H-HeJ *male* mice was treated exactly like the Cohort 3 mice.

#### Isolation of sEVs from tissue explants

EVs from tumors in Cohort 3 were isolated as follows. Each tumor was cut into small pieces, which were placed in a well of the 6-well plate and cultured in RPMI medium (5 ml) supplemented with 1% (v/v) penicillin/streptomycin at 37°C for 48 h. Supernatants containing EVs were collected by aspiration, clarified by centrifugation at 2000 × *g* for 10 min to sediment cell fragments and then transferred to new tubes for centrifugation at 10 000 × *g* at 4°C for 30 min to sediment microvesicles (MVs). The supernatants were collected and filtrated using a 50 ml syringe and a 0.22 µm bacterial filter. The final precleared supernatants were used for SEC to isolate sEVs as described above.

#### sEV isolation from murine blood

Blood collected in heparinized tubes was centrifuged at 2000 × *g* for 10 min, and then at 10 000 × *g* for 30 min. The preclarified plasma was diluted 5× with PBS and filtered (0.2 µm filter), and 100 µl of filtered plasma was applied to a small Bio-spin Chromatography Column (Bio-Rad #7326008) packed with Sepharose 2B for sEV isolation by SEC. PBS was used for elution, and 200 µl fractions were collected. The second fraction (#F2) containing majority of the vesicles was collected for single EV flow cytometry using Cytoflex.

#### sEV counts by single sEV flow cytometry

The concentration and size of the EVs in the preclarified plasma before SEC were analyzed by NTA. For flow cytometry by Cytoflex (Beckman Coulter, Pasadena, CA, USA), the vesicles in #F2 were incubated with Di-8-Anneps dye (final concentration: 0.5 µM, Biotium) in the presence of 0.01% Pluronic F-127 (ThermoFisher Scientific) for 1 h at RT in the dark. The dye-labeled vesicles were diluted (4–16×) in PBS before performing single EV flow cytometry. The dye-labeled vesicles were detected in the PerCp channel, the gain was set at 200 for FSC, SSC, and vSSC channel, and threshold for PerCp height channel was set at >1500. Samples were run at 60 μl/min for 2 min. The first 30-s runs were discarded, and remaining 90-s runs were used for analysis. The flow cytometry data were analyzed using FlowJo software.

#### Histology and immunohistochemistry

Tissues were processed for paraffin sections following fixation using routine methods and paraffin embedded. Sections were stained with hematoxylin and eosin (H&E), examined, and imaged at the Research Histology Core Laboratory (TARPS) (http://pittbiospecimencore.pitt.edu). Excised implants/tissue samples were placed in 4% paraformaldehyde for 24 h, and subsequently in 30% sucrose (Sigma-Aldrich) for 24 h. Samples were embedded in OCT (ThermoFisher Scientific) and stored at −80°C for subsequent sectioning. Cryostat sections (6 μm) were cut, and immunofluorescence staining was performed at the Research Histology Core Laboratory (TARPS). Tissue sections were incubated with primary Abs overnight at 4°C. After extensive washing, tissue sections were incubated with secondary Abs at RT for 20 min followed by PBS washing ×3. Negative controls were stained in parallel with the secondary Abs alone. Sections were counterstained and mounted with DAPI Fluoromount-G® (Southern Biotech) and imaged using a fluorescence microscope.

### Statistical analysis

Unpaired Student’s *t*-test was used to compare the independent samples between two groups. The data are presented as means ± SD of three independent experiments. One-way analysis of variance (ANOVA) with *post hoc* test (Tukey’s test) was used when multiple comparisons were performed. Graph Pad Prism 10.3 was used for graph drawing and statistical analysis. *P* values <0.05 were considered significant.

## Results

### *In vitro* effects of AMD on cell growth and TEX production, phenotype, and molecular content

Based on a report (24) that acute elevation of Ca^2+^ in cancer cells stimulates release of EVs, we asked whether the inhibition of Ca2^+^ channels using AMD could block TEX secretion by cultured tumor cells. We found that plated SCCVII cells (1 × 10^6^) released from 3.5 × 10^9^ to 5.5 × 10^9^ TEX in 48 h. TEX harvested from tumor cell supernatants by SEC had vesicular morphology, were sized at 50–200 nm (median = 105 nm), and carried CD9, TSG101, and ALIX but were negative for calnexin (Supplementary Fig. 1a). These TEX were readily taken up and internalized by autologous tumor cells (Supplementary Fig. 1b). Further, these TEX added to culture medium in place of serum promoted tumor cell proliferation (Supplementary Fig. 1c) by autocrine stimulation.

**Figure 1:**
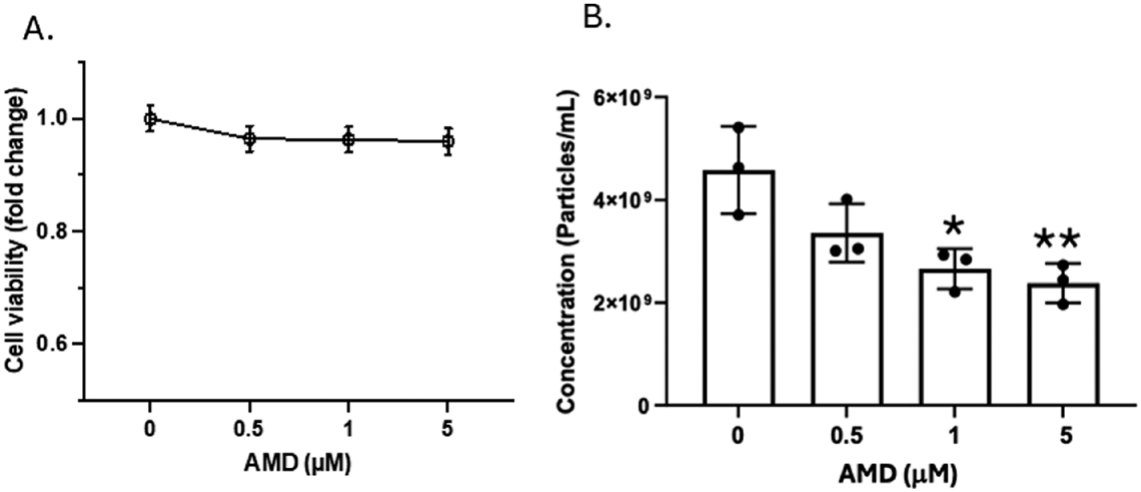
*In vitro* effects of AMD on the viability and numbers of cultured SCCVII cells. (a) Using MTT assays, no change in the tumor cell viability relative to increasing AMD concentrations was evident after 48-h culture. (b) Changes in the numbers of TEX produced by tumor cells in the presence of various AMD concentrations as measured by NTA after 48-h culture. Data are mean values ± SD from triplicate cultures. *P < 0.03; **P < 0.02 for AMD treated versus untreated. Unpaired *t*-test was used for statistical analysis.

Next, murine SCCVII cells and human HNSCC cell lines (PCI-13, SSC47) were cultured in the absence/presence of various AMD concentrations (10–50 µM) to evaluate potential AMD toxicity for tumor cells. In human PCI-13 cells cultured at increasing AMD concentrations for 48 h, cell viability was reduced to 90% of the DMSO control, and at 20 µM of AMD, it was reduced to 50% of control (data not shown). The experiments indicated that at AMD concentrations lower than 10 µM, tumor cell viability was unimpaired. For SCCVII cells, grown in medium containing 1–5 µM AMD for 24–48 h, cell viability remained at 100% of control (Fig. 1a). Supernatants of SCCVII cells cultured in the presence of 0.5–5.0 µM AMD for 48 h were tested for TEX numbers by NTA (Fig. 1b). A significant AMD concentration-dependent decrease in TEX numbers was consistently observed. Similar effects of AMD on TEX numbers were observed with human HNSCC cell lines, PCI-13 and SCC47.

**Figure 2:**
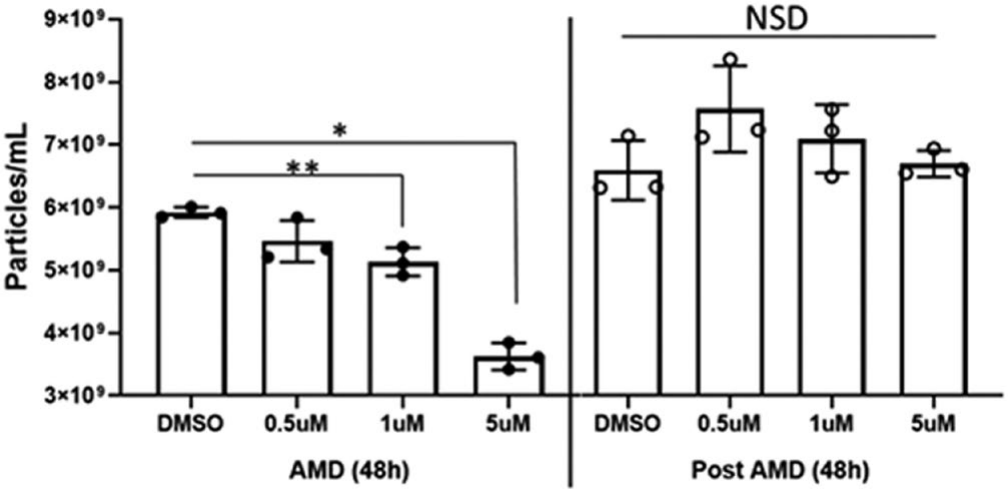
AMD reduced the numbers of TEX produced by cultured PCI-13 cells (*left*). Data are mean NTA numbers ± SD from triplicate cultures. *P < 0.00 and **P < 0.005. NSD = no significant difference. Note that the y-axis does not start at 0. After AMD replacement with fresh medium and 48-h culture *(right*), the numbers of TEX rebound to numbers seen in untreated controls. Unpaired *t*-test was used for statistical analysis.

Figure 2 illustrates changes in numbers of TEX recovered from supernatants of PCI-13 cells treated with increasing AMD concentrations. As AMD concentrations increased, significant decreases in TEX release from tumor cells were observed in 48-h cultures. When AMD-containing medium was removed and replaced with fresh medium, numbers of TEX released by PCI-13 cells rebound to those in untreated controls and remained unchanged in all cultures. A similar rebound in TEX numbers after AMD removal was observed in the other HNSCC cell line, SCC47, and in murine SCCVII cells (data not shown).

In aggregate, these studies indicated that *in vitro* treatment of human or murine tumor cells with increasing AMD concentrations (range, 0.5–5.0 µM) for 48 h did not impair cell growth but significantly reduced TEX release from tumor cells into the supernatant. Further, after AMD was removed and replaced by fresh medium, the numbers of TEX released by tumor cells rebounded to that of untreated controls within 48 h, suggesting that the continued presence of AMD is required for the inhibition of TEX release by tumor cells.

**Figure 3:**
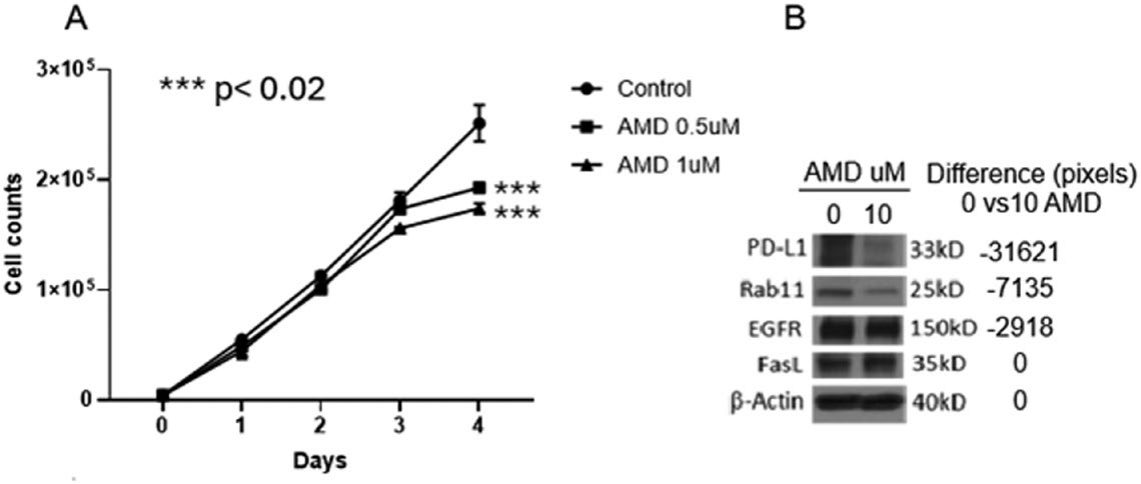
Effects of AMD on *in vitro* proliferation and the protein content of SCCVII tumor cells. (a) AMD concentration-dependent and time-dependent inhibition of tumor cell growth. Data are mean values ± SD from triplicate cultures. Unpaired *t*-test was used for statistical analysis. (b) Western blots of SCCVII cell lysates. Cells were incubated with AMD or DMSO in buffer for 24 h. Lysates of equivalent cell numbers were prepared and aliquots containing 10 µg total protein were applied to each lane for protein electrophoresis. Note that lysates of tumor cells treated with 10 µM AMD for 24 h contained lower levels of PD-L1 and Rab11.

AMD not only reduced numbers of TEX released by tumor cells but also significantly decreased tumor cell proliferation (Fig. 3a) and selectively altered the TEX protein content. Effects of AMD on tumor cell proliferation were concentration and time dependent, and became significant only after 72-h culture. Also, AMD altered the cellular content of PD-L1 and Rab11 proteins in tumor cells, suggesting that the expression of proteins associated with immune regulation (PD-L1) and TEX secretion (Rab11) was affected.

**Figure 4:**
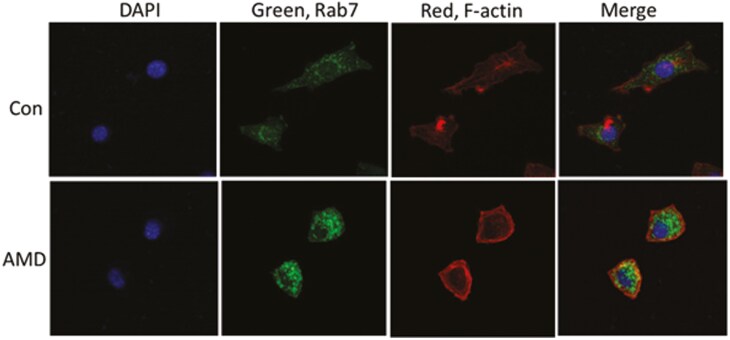
Immunofluorescence images of SCCVII cells indirectly stained with anti-Rab7 Abs (green) showing accumulations of granules in the cytoplasm of AMD-treated tumor cells and with F-actin rhodamine-falloidin (red) to illustrate changes in the cell shape. Confocal microscopy: magnification x400

Further, upon coincubation of SCCVII cells ± AMD (2 µM for 6 h), the tumor cell size and shape were altered relative to PBS controls (Fig. 4). Staining of AMD-treated tumor cells with anti-Rab-7 Abs showed accumulations of numerous large aggregates of labeled vesicles in the cytosol. In contrast, control cells (no AMD) contained fewer TEX situated in the perinuclear region of tumor cells (Fig. 4). The images of AMD-treated SCCVII cells suggested that AMD affects the vesiculation process, perhaps by preventing the release of vesicles and enhancing their accumulation in the cytosol.

In aggregate, the data in Figs 1–4 suggest that tumor-derived EVs are necessary for support of tumor growth and that by inhibiting EVs production/release, AMD interfered with tumor cell growth. The effects of AMD also include alterations in the protein content of tumor cell lysate and in the shape and size of tumor cells. Simultaneous accumulation of Rab7^+^ granules in the cytosol of AMD-treated tumor cells (Fig. 4) indicates that AMD interferes with EV release into the extracellular space, thus preventing EV reutilization by the tumor. Together, the data suggest that tumor-derived EVs mediate autocrine effects which favor growth of the autologous tumor.

**Figure 5:**
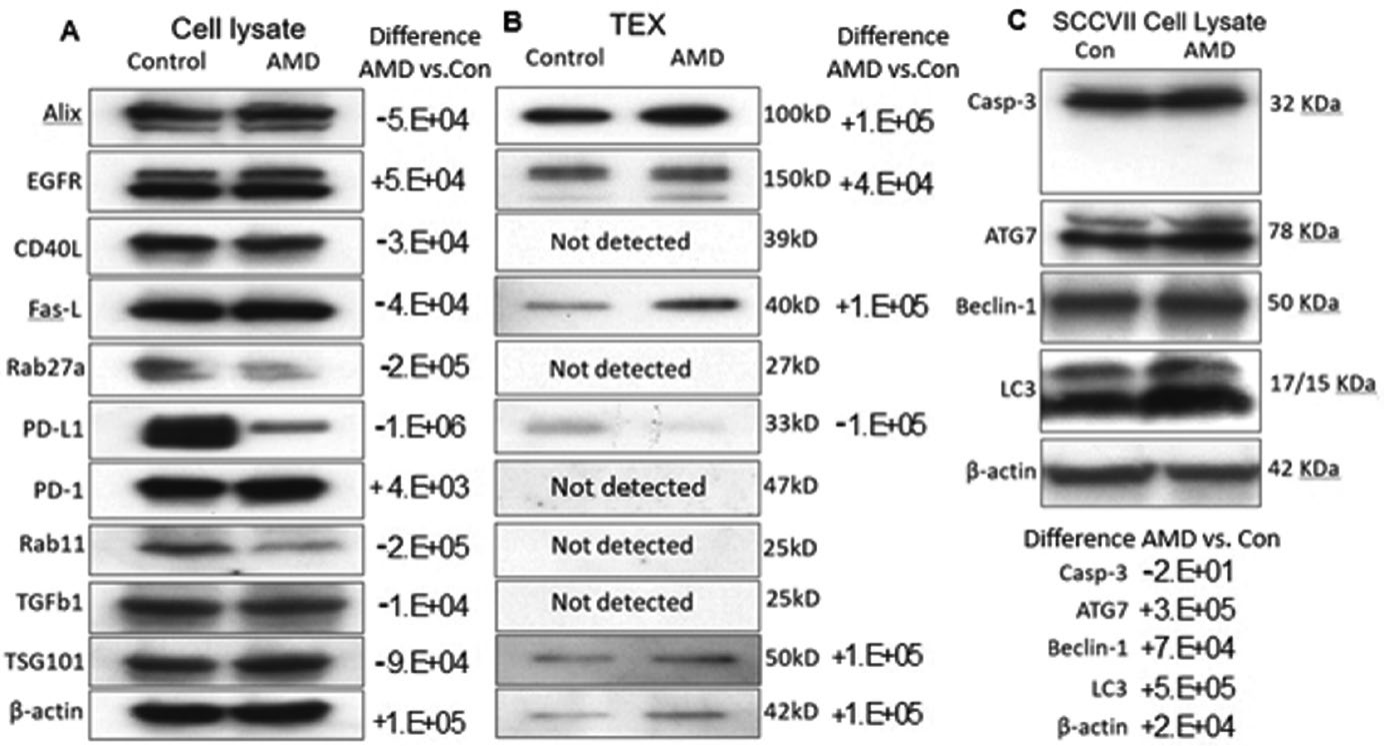
Protein content in tumor cell lysates ± AMD and paired TEX. (a) Effects of AMD (2.5 µM, 24 h) on expression of selected proteins in SCCVII lysates. Tumor cells were treated with PBS as control for 24 h. (b) WBs of TEX isolated from the paired cell supernatants. Protein expression in lysates of cells and paired TEX produced by these cells were compared in WBs. Aliquots containing 10 µg total protein were applied to each lane for protein electrophoresis. (c) WBs showing the upregulation of expression levels of proteins mediating autophagy, ATG7, Beclin-1, and LC3, in SCCVII cells treated with AMD (5.0 µM, 24 h). For (a–c), representative WBs selected from three independent experiments are shown.

To further explore changes in the protein profiles of AMD-treated SCCVII cells, additional WBs were performed with lysates of SCCVII incubated in the presence/absence of AMD. Figure 5a shows that AMD decreased expression levels of Rab11, Rab-27, PD-L1, and TGF-β1 but not EGFR or CD40L. Expression levels of ALIX, TSG101, PD-1, and FasL increased in SCCVII cells treated with 2.5 µM AMD for 24 h. Thus, the protein profile of SCCVII cells was altered following treatments with AMD, and the protein alterations observed were associated with the vesiculation process and immune regulation. As TEX generally mimic the molecular content of parent cells, we expected that the protein content of TEX produced by AMD-treated SCCVII cells would differ from that in TEX produced by PBS-treated SCCVII control cells. WBs of TEX paired with lysates of tumor cells that had produced these TEX are shown in Fig. 5b. TEX produced by AMD-treated SCCVII cells show an enrichment in ALIX, TSG101, and FasL and notably minimal expression of PD-L1. There was an absence of Rab11 and Rab27a in EVs from cells treated with PBS or AMD.

To assess whether AMD activated autophagy in SCCVII cells, lysates of SCCVII cells incubated with AMD (5 µM) or PBS for 24 h were tested by WBs for the evidence of upregulated expression of ATG7, Beclin-1, and LC3, the proteins known to mediate autophagy (Fig. 5c). The quantitative WB data show that AMD used at concentrations of 2.5–5.0 µM increased expression levels of these proteins and did not induce caspase 3 activation (Fig. 5c). These preliminary results suggest that AMD promoted autophagy in tumor cells which was induced by autocrine signals driven by TEX and did not involve extrinsic apoptosis of tumor cells. This finding suggests that tumor cell-derived EVs are necessary for support of tumor growth and survival, potentially by promoting autophagy, and that TEX are not suppressive or cytotoxic to the autologous tumor.

### *In vivo* effects of AMD on SCCVII growth and TEX production

In the initial *in vivo* experiments, normal immunocompetent 8-week-old female CH3H/HeJ mice (Cohort 1) were injected IP with PBS or AMD at 10 mg/kg daily for 15 days, and numbers of EVs in plasma were measured using flow cytometry prior to AMD and once weekly after AMD treatments. Supplementary Fig. 2 shows that the EV numbers in plasma of mice receiving 10 mg/kg of AMD decreased relative to PBS controls, albeit variably and not significantly.

The experiment was repeated using 15 mg/kg of AMD instead of 10 mg/kg. At this AMD dose, the EV numbers in plasma were reduced but again variably and not significantly, largely because of the count variability in individual mice. However, at this dose of AMD, the mice became agitated and aggressive, suggesting that this dose of AMD may interfere with well-being of the animals. Based on these results, the AMD dose of 10 mg/kg was selected for all subsequent *in vivo* experiments.

**Figure 6:**
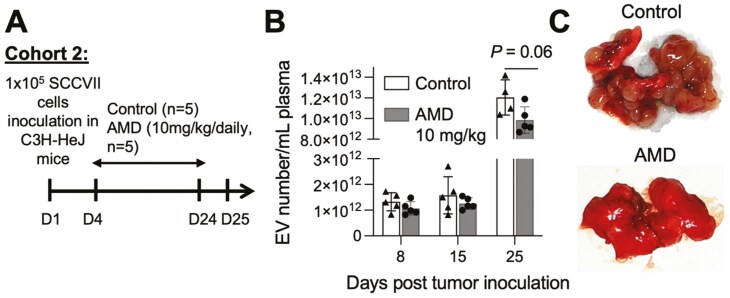
Effects of AMD on the EV numbers in plasma of mice bearing lung metastases. (a) Schema of the study timeline. (b) Numbers of EVs were measured by NTA in plasma of SCCVII-bearing C3H-HeJ mice receiving daily AMD (IV, 10 mg/kg) or vehicle control starting on day 4 after the tumor implantation. Note the increase in EV numbers as the tumor grows. Data presented as mean numbers ± SD. Unpaired *t*-test was used for statistical analysis (c). Representative lung specimens selected from the control #2 mouse and AMD-treated #4 mouse. Note the presence of numerous lung metastases in the control lung and only few metastases in the lung of AMD-treated mouse.

To assess the effects of AMD in tumor-bearing mice, SCCVII cells (1 × 10^5^) were injected IV via a tail vein in C3H-HeJ mice (Cohort 2; *n* = 5), and daily AMD injections were started on day 4 post-tumor injection (Fig. 6a). In these mice, lung metastases are visible within 14 days. Control mice received PBS. The mice were sacrificed on day 25; lungs were harvested and examined for the presence of metastases. The numbers of EVs in plasma were measured serially by NTA. Figure 6b shows that numbers of EVs in plasma increased as the tumor grew and metastasized. The plasma EV numbers in AMD-treated mice were lower than those in control mice, but the difference was not significant. The numbers of lung metastases in AMD-treated mice were fewer than in control mice but could not be precisely quantified because numerous metastatic nodules tended to coalesce.

**Figure 7:**
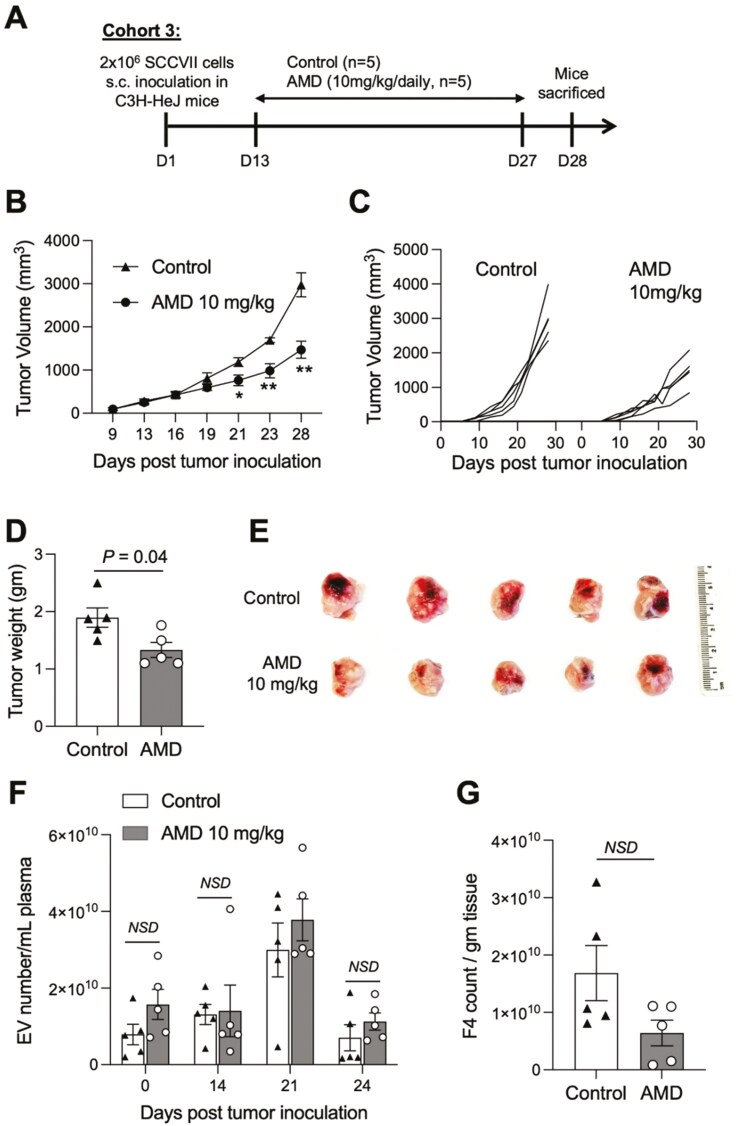
Effects of AMD on growth of established SCCVII tumors in female C3H-HeJ mice (Cohort 3). (a) Schema of the study timeline. Mice received daily IP injections of AMD or vehicle as control. (b, c) In the tumor-bearing mice treated with AMD, tumor volume was significantly reduced compared to that in control mice. *P < 0.05, **P < 0.001. (d) Tumor weights at the time of sacrifice on day 27 were also significantly reduced, and tumor sizes were smaller in AMD-treated versus control mice (e). (f) The total sEV numbers/mL plasma were variable and were not decreased in the AMD-treated mice relative to controls when single vesicles were counted by flow cytometry (Cytoflex). (g) Numbers of sEV/gram tumor tissue that eluted from excised tumors tends to be lower in AMD-treated mice compared to controls (*P* = 0.08). Data are mean values ± SD. Unpaired *t*-test was used for statistical analysis.

These results suggested that daily AMD treatment (10 mg/kg) of tumor-bearing mice for 21 days decreased numbers of lung metastases and concomitantly tended to decrease the numbers of EVs in plasma, although neither of these effects achieved statistical significance. These preliminary data suggested that AMD delivery to tumor-bearing mice might inhibit EV production by the tumor and might suppress tumor metastasis formation. However, assessments of the size and numbers of metastases in this lung model of SCCVII were unreliable. Therefore, we resorted to a subcutaneous model of SCCVII established by injecting 2 × 10^6^ tumor cells in the right flank of each mouse. In this model, changes in tumor growth and plasma EV numbers during AMD delivery could be readily and reliably monitored. As shown in Fig. 7, in the Cohort 3 (C3H-HeJ; *n* = 10), tumor cells were injected sc on day 0. Daily AMD delivery (IP) was commenced on day 13, when the tumor was palpable, and it continued until the animals were sacrificed on day 28.

The data obtained with the Cohort 3 illustrate effects of AMD on tumor growth and on numbers of sEV in plasma and in tumor tissues. Daily IP delivery of AMD at 10 mg/kg to the tumor-bearing mice slowed tumor growth. Tumor volumes at the time of sacrifice (day 27) were significantly reduced. Tumor weights were also significantly decreased in mice treated with AMD versus controls (Fig. 7d). The weights and sizes of excised tumors were likewise significantly smaller in the AMD-treated mice (Fig. 7e). The total sEV numbers in the plasma were not decreased in the AMD-treated mice (Fig. 7f). However, examination of EVs that eluted from excised tumor tissues showed that the numbers of sEV/gram of tumor tissue tended to decrease (Fig. 7g). The data suggested that AMD selectively influenced the production level/release of tumor-derived EVs, i.e. TEX, and had broadly variable effects on numbers of total plasma sEVs. As EV numbers in plasma are a sum of TEX and non-TEX, the variable numbers of EVs in plasma likely reflect differences in the TEX content, which is different in each mouse.

**Figure 8:**
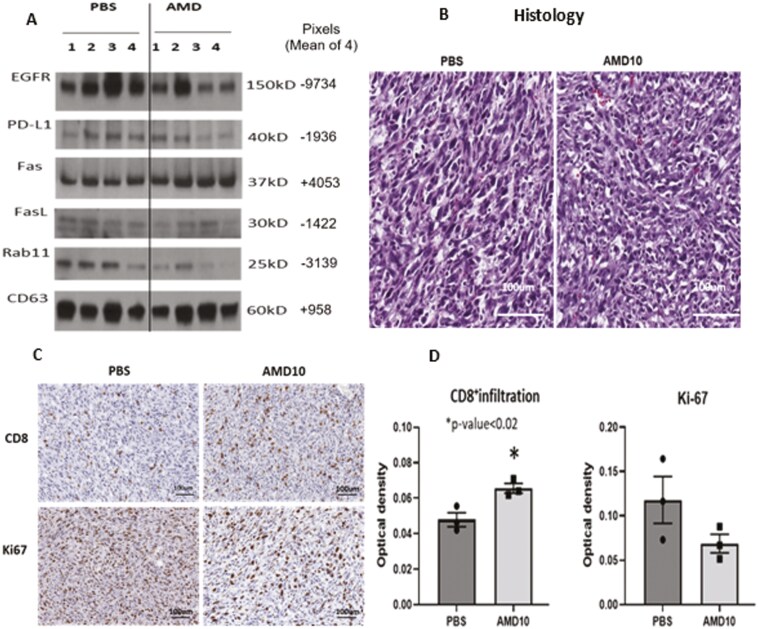
The altered protein content of EVs eluted from tumor explants and immunopathology of tumor tissues from AMD-treated mice. (a) WBs of EVs eluting from excised tumor tissue into supernatants. EVs were isolated by SEC and EV lysates were analyzed by WBs for selected proteins (20 µg protein loaded/gel). Shown are representative WBs for EVs eluted from tumors of individual PBS-treated (*n* = 4) and AMD-treated (*n* = 4) mice from the Cohort 4. (b) Histopathology (H&E) of SCCVII tumors obtained from mice (Cohort 4) treated with AMD (10 mg/kg) or PBS. Note distorted cellular morphology of the tumor excised from an AMD-treated mouse. (c) Infiltration of the tumor with CD8^+^ T cells ± AMD and staining for Ki67 in tumor tissue ± AMD. (d) Quantitative results (means ± SD) for staining of tumor sections obtained from three animals for CD8^+^ cells (*P* < 0.02) or Ki67 (*P* = 0.15). Unpaired *t*-test was used for statistical analysis.

Next, to evaluate potential effects of gender on response of SCVII tumors to AMD, the above-described Cohort 3 experiment using female mice was repeated with male mice (Cohort 4). In this experiment, we included 12 mice treated with 10 mg/kg of AMD, 12 mice treated with 15 mg/kg, and 12 control mice (Supplementary Fig. 3). The results of AMD effects on tumor growth and sEV numbers in plasma reproduced the data reported above for Cohort 3 mice. In addition, dissected, minced tumors were incubated in serum-free medium for 48 h, and sEVs eluted from the excised tumor tissue were evaluated by WBs for the content of selected proteins that potentially could modulate vesicular functions (Fig. 8a). The EVs eluting from tumors of the AMD-treated mice had lower levels of PD-L1 and Rab11, while levels of CD63, EGFR, or Fas were not different from PBS controls. These data were similar to the phenotype of EVs produced by tumor cells treated with AMD *in vitro* (Fig. 3). The histopathology of tumors from mice treated with AMD (Cohort 4; see Supplementary Fig. 3) showed grossly dysregulated cellular morphology (Fig. 8b). Immunostaining of tumor tissues with anti-Ki67 Abs indicated that tumors from AMD-treated mice had fewer Ki67^+^ cells than controls (Fig. 8c). Importantly, infiltration of CD8^+^ T cells into the tumor tissue was significantly increased in AMD-treated mice relative to untreated controls (Fig. 8d).

In aggregate, the results of *in vivo* experiments have led to the conclusion that the delivery AMD to female or male CH3-HeJ mice interfered with the production of TEX and decreased their release, preventing or decreasing TEX reutilization by the tumor and resulting in suppression of tumor growth.

## Discussion

In this work, we showed that the L-type calcium channel inhibitor, AMD, inhibited growth of murine and human tumors in culture and of murine SCCVII *in vivo*. These growth inhibitory effects were associated with the reduction in numbers of TEX released by SCCVII cells, alterations in immunoregulatory proteins carried by TEX and in enhanced infiltration of the tumor by CD8(+) T cells. Changes were seen in the tumor cellular architecture and the ability of tumor cells to migrate, as judged by a loss of fibroblast-like morphology. By disrupting TEX production/release from tumor cells, AMD interfered with the tumor-TEX autocrine pathways, including autophagy, which are essential for tumor survival. Concomitantly, numbers of tumor-infiltrating immune effector cells increased, presumably contributing to suppression of the tumor growth.

AMD, a canonical calcium channel inhibitor, is widely used as treatment for lowering blood pressure (25,26). Available data confirm the major role of intracellular Ca^2+^ in regulated exocytosis in most cell types, including tumor cells (24,27,28). Data show that accumulation of intracellular Ca^2+^ in the multivesicular bodies (MVBs) stimulates sEV secretion (29). Mechanistically, the membrane of a secretory vesicle (MVB) fuses with the plasma membrane in a tightly controlled Ca^2+^-triggered reaction that regulates vesicular release (29). Acute elevation of Ca^2+^ levels in tumor cells is a universal intracellular signal for regulated vesiculation/secretion (30). The elevation of Ca^2+^ levels in cancer cells stimulated a 5-fold increase in release of CD63^+^CD9^+^ALIX^+^ exosomes (28). This finding provides a plausible explanation for why tumor cells produce and release elevated numbers of vesicles relative to nonmalignant cells and why plasma of cancer patients contains elevated EV numbers (14,31). A recent study reported that AMD induced Ca^2+^-dependent PD-L1 degradation and promoted rejuvenation of anti-tumor responses in colorectal MC38 tumor model established in mice. AMD not only suppressed PD-L1/PD-1 binding and augmented T-cell cytotoxicity *in vitro* and *in vivo*, but also inhibited tumor growth and enhanced efficacy of anti-PD-L1 therapy in mice (19). The authors suggested that AMD could be repurposed as a drug regulating tumor growth and potentiating anti-tumor activity of T cells in subjects with cancer (19).

This study evaluated the potential role of AMD in autocrine regulation of TEX production/release by murine SCCVII (and by human solid tumors *in vitro*). Tumors are dependent on autocrine release and reutilization of their metabolic contents for survival in a highly unfriendly microenvironment (32,33). Vesiculation is an acknowledged mechanism of tumor growth promotion (34,35), and interference of AMD with vesiculation and reutilization of TEX by autologous tumor impairs its growth (19,35). AMD reduced numbers of TEX produced and released by the tumor, thereby reducing TEX availability for support of tumor growth. AMD also appears to enhance or restore anti-tumor functions of immune cells in tumor-bearing mice (19). In our study, AMD delivered to tumor-bearing hosts simultaneously inhibited TEX release, altered TEX molecular content to a less immunosuppressive phenotype, and restored anti-tumor immune functions, providing a compelling rationale for repurposing AMD as a cancer drug. Also, AMD appears to be a *selective* inhibitor of TEX release which does not interfere with vesiculation in nonmalignant cells/tissues. The latter is necessary for maintaining EV-mediated intercellular communication. A selective blockade of immunosuppressive TEX (8) is a highly attractive approach to disrupting tumor-promoting cross-talk that TEX mediate.

Our preliminary *in vitro* results indicate that molecular underpinnings of AMD-mediated blockade of TEX release include activation of autophagy in tumor cells. Induction of excessive autophagy is considered a major factor in stress-induced tissue damage occurring during myocardial infarction, stroke, or organ transplantation (36). In cancer, inhibition or stimulation of autophagy is linked to alterations in EV biogenesis and release (37). Specifically, knock out (KO) of autophagy proteins ATG12-ATG-3 concomitantly reduced the intraluminal vesicle (ILV) formation in MVBs and EV secretion (38). Further studies are in progress to more precisely define the molecular mechanisms regulating effects of AMD on autophagy *vis a vis* TEX secretion by SCCVII tumor cells in support of the potential role of AMD as anti-cancer drug. In our hands, low dose-dependent continuous AMD delivery to mice with progressing tumors was therapeutically effective. AMD delivered daily to mice at doses higher than 10 mg/kg induced anxiety and behavioral problems, while its prolonged daily delivery at 10 mg/kg was well tolerated and effective in blocking TEX release by the growing tumor. This represents another potential advantage of AMD as a cancer drug which could be readily combined with other therapies, including immunotherapy. Many patients with cancer are taking Ca^2+^ channel inhibitors for control of blood pressure, and repurposing of AMD as cancer therapy in these patients would be seamless from the regulatory point of view. However, it is possible that other CCBs widely used for controlling hypertension, e.g. nifedipine or felodipine, could also block calcium channels in tumor cells. Thus, previous treatment of cancer patients with these CCBs, including AMD, would be likely to lower responsiveness to the AMD therapy. If this proves to be the case, the AMD therapy might not be effective in patients routinely treated with CCBs or might require previous suspension of the CCB therapy. Further *in vivo* studies in mouse tumor models are indicated to further elucidate potential interference of other CCBs with AMD therapy.

## Supplementary Material

bgaf016_suppl_Supplementary_Figures

## Data Availability

The data generated in this study are available upon request from the corresponding author.
