# Supplementary material for: Amlodipine, an L-type Ca2+ channel inhibitor, regulates release of extracellular vesicles from tumor cells
Source: Carcinogenesis. 2025 Mar 23;46(2):bgaf016. doi: 10.1093/carcin/bgaf016 (PMC12096003; doi:10.1093/carcin/bgaf016)
Supplement: bgaf016_suppl_Supplementary_Figures [file bgaf016_suppl_supplementary_figures.pdf]

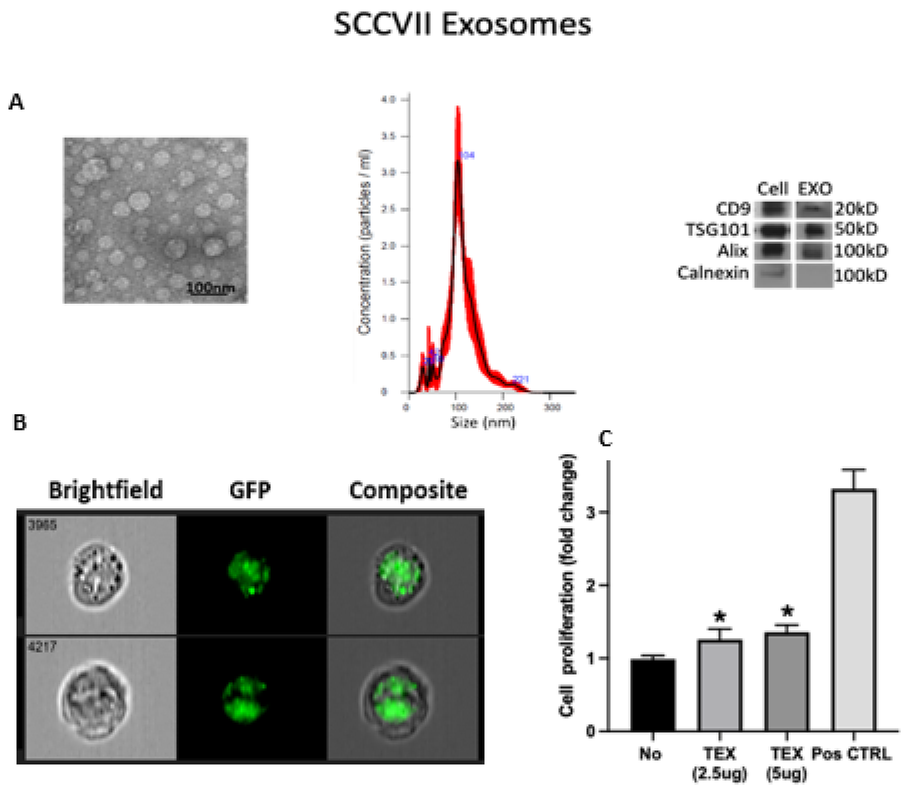

SFigure 1. Characteristics of TEX produced by cultured SCCVII cells. In (**A**), TEX morphology by transmission electron microscopy; TEX size and concentration as measured by NTA; WBs of tumor cell lysate and isolated TEX In (**B**), TEX produced by SCCVII cells were transfected with CD63-GFP by nucleofection and were co-incubated with unlabeled SCCVII cells for 12h. TEX were internalized and are seen inside the tumor cells. In (**C**), autocrine effects of TEX produced by SCCVII cells and added to SCCVII cultured in media without serum (No) for 24h. After additional 48h, proliferation of tumor cells ± TEX was measured. Pos CTR = tumor cells in medium containing 10% (v/v) FBS. Data are mean values ± SD from 3 experiments. * P<0.05.

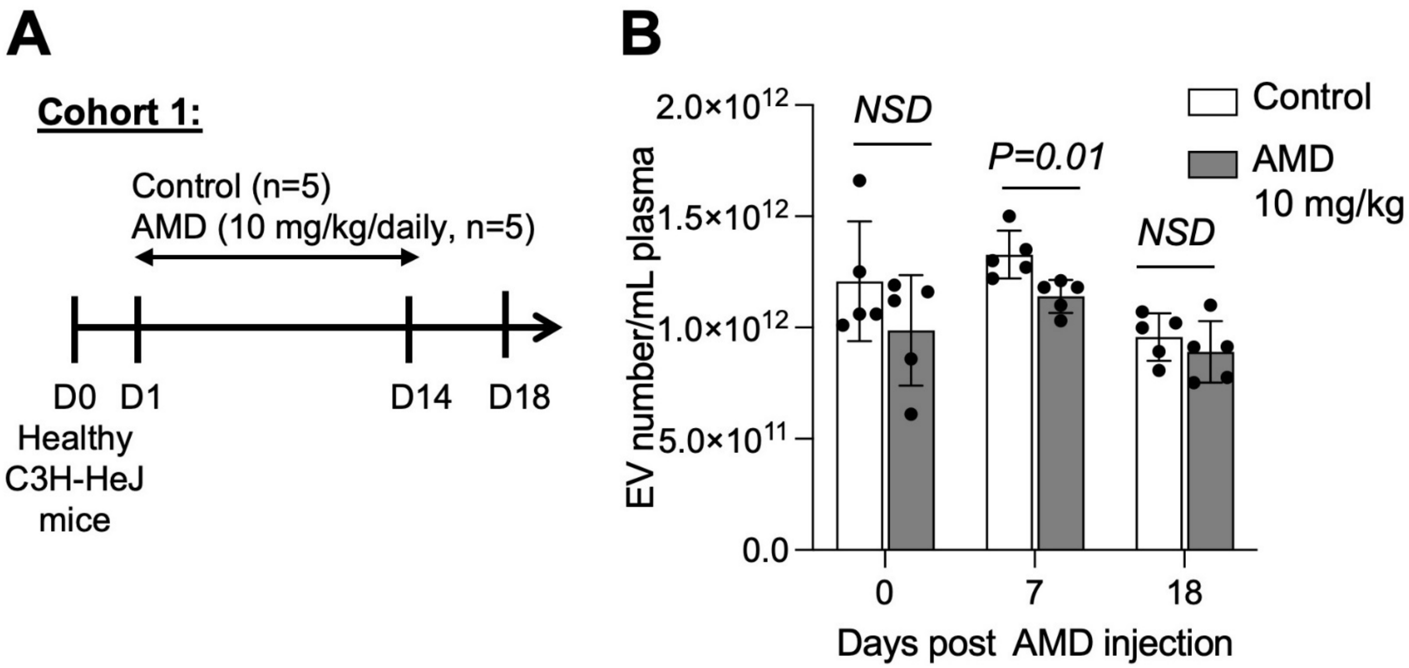

SFigure 2. **Numbers of EVs in plasma of healthy C3H-HeJ mice (n=5) treated with 10mg/kg AMD or with PBS as control.** Numbers of EVs in plasma were measured prior to AMD and after AMD delivery on day 18. Data are mean values ± SD. Statistical analysis was performed using unpaired t-test. NSD= no significant difference.

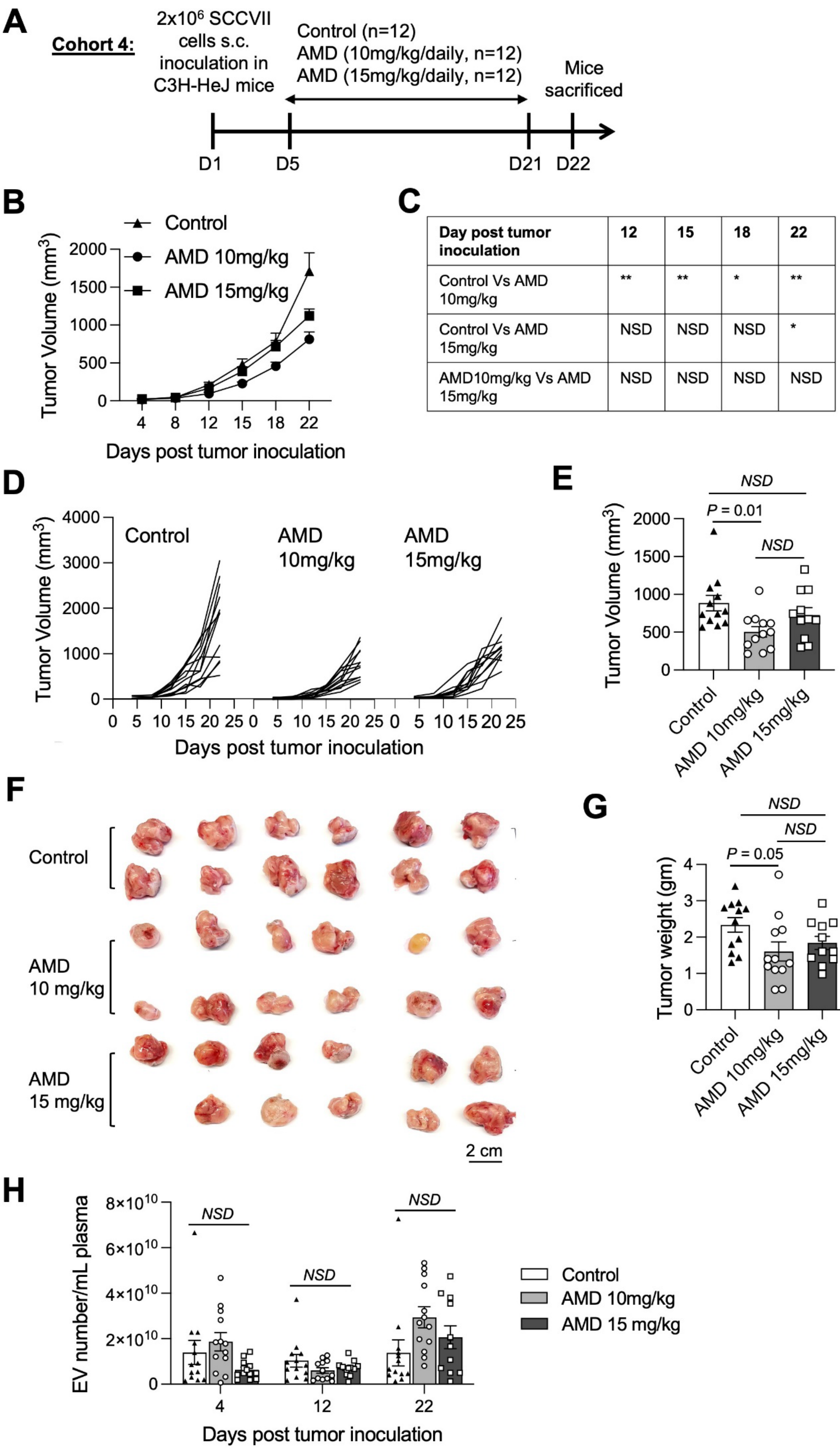

Day post tumor inoculation	12	15	18	22
Control Vs AMD 10mg/kg	**	**	*	**
Control Vs AMD 15mg/kg	NSD	NSD	NSD	*
AMD10mg/kg Vs AMD 15mg/kg	NSD	NSD	NSD	NSD

SFigure 3. Treatment of C3H-HeJ mice (**Cohort #4**) bearing subcutaneous SCCVII tumors with AMD. In (**A)**, experimental design for treatment and blood draws. In (**B)**, combined tumor volumes for all mice treated with AMD (10mg/kg or 15mg/kg) or with vehicle. In (**C)**, the table shows results of multiple comparisons using one-way analysis of variance (ANOVA) with Tukey's multiple comparisons: *P<0.01; **P<0.001. In (**D)**, tumor volumes in individual mice treated with AMD (10mg/kg) or with vehicle. In (**E)**, volume of excised tumors (mean values ± SD). In (**F)**, images of excised tumors. In (**G)**, tumor weights (mean values ± SD). In (**H)**, numbers of EVs in plasma of mice treated with AMD or vehicle determined by single-EV flow cytometry using Cytoflex. The statistical differences were calculated using one-way ANOVA with Tukey's post hoc test.

STable 1. Antibodies used for WBs, and Immunofluorescence or Immunochemistry.

Antibody	Supplier	Catalog #
ATG7AB	Santa Cruz	SC-7148
Alix Ab	Thermo Fisher	MA5-32773
Beclin-1 Ab	Santa Cruz	SC-11427
Beta actin Ab	Santa Cruz	SC-47778
Caspase-3 Ab	Santa Cruz	SC-7148
CD40L Ab	Thermo Fisher	MA5-32619
Calnexin Ab	Cell Signaling	24335
CD9 Ab	Cell Signaling	131745
CD63 Ab	Abcam	217345
CD8 Ab	Invitrogaen	MA5-17602
F-actin (Rhodamine phalloidin)	Invitrogen	R415
Fas Ab	Biolegend	152603
Fas L Ab	Cell Signaling	72062
KL67 Ab	Invitrogen	740008TB
LC3 Ab	Cell Signaling	4108
PD-1 Ab	Cell Signaling	84651
PD-L1 Ab	Abcam	AB213480
Rab7 Ab	Abcam	137029R
Rab11 Ab	Cell Signaling	3539
Rab27a Ab	Cell Signaling	95394
TGFβ1 Ab	Abcam	AB31013
TSGI01 Ab	Abcam	AB83
